# Maternal Dietary Fiber Composition during Gestation Induces Changes in Offspring Antioxidative Capacity, Inflammatory Response, and Gut Microbiota in a Sow Model

**DOI:** 10.3390/ijms21010031

**Published:** 2019-12-19

**Authors:** Yang Li, Haoyu Liu, Lijia Zhang, Yi Yang, Yan Lin, Yong Zhuo, Zhengfeng Fang, Lianqiang Che, Bin Feng, Shengyu Xu, Jian Li, De Wu

**Affiliations:** Key Laboratory for Animal Disease-Resistance Nutrition of the Ministry of Agriculture, Animal Nutrition Institute, Sichuan Agricultural University, Huimin Road 211#, Chengdu 611130, China; liyang_cc@yeah.net (Y.L.); liuhy_sicau@163.com (H.L.); Zhang-Lijia@outlook.com (L.Z.); yangyi@stu.sicau.edu.cn (Y.Y.); Linyan936@163.com (Y.L.); zhuoyong@sicau.edu.cn (Y.Z.); fangzhengfeng@hotmail.com (Z.F.); clianqiang@hotmail.com (L.C.); fengbin@sicau.edu.cn (B.F.); shengyu_x@hotmail.com (S.X.); lijian522@hotmail.com (J.L.)

**Keywords:** fiber, gut microbiota, inflammation, maternal nutrition, offspring, oxidative stress

## Abstract

To study the effects of maternal dietary fiber composition during gestation on offspring antioxidant capacity, inflammation, and gut microbiota composition, we randomly assigned 64 gilts to four treatments and administered diets with an insoluble/soluble fiber ratio of 3.89 (R1), 5.59 (R2), 9.12 (R3), and 12.81 (R4). Sow samples (blood and feces at gestation 110) and neonatal samples (blood, liver, and colonic contents) were collected. The results showed that sows and piglets in R1 and R2 had higher antioxidant enzyme activity and lower pro-inflammatory factor levels than those in R3 and R4. Moreover, piglets in R1 and R2 had higher liver mRNA expression of Nrf2 and HO-1 and lower NF-κB than piglets in R4. Interestingly, maternal fiber composition not only affected the production of short-chain fatty acids (SCFAs) in sow feces but also influenced the concentrations of SCFAs in the neonatal colon. Results of high-throughput sequencing showed that piglets as well as sows in R1 and R2 had microbial community structures distinct from those in R3 and R4. Therefore, the composition of dietary fiber in pregnancy diet had an important role in improving antioxidant capacity and decreasing inflammatory response of mothers and their offspring through modulating the composition of gut microbiota.

## 1. Introduction

Reactive oxygen species (ROS), such as superoxide and hydrogen peroxide, are constantly generated from oxygen in all aerobic metabolism and pathogenic processes [1]. Oxidative stress occurs when the balance between the generation of ROS and the antioxidant defense capacity of the body is destroyed [2]. Oxidative stress can lead to a cascade of reactions that damage lipids, proteins and/or DNA, and cause a number of human diseases [3]. During the neonatal period, ROS play an important role in the onset of many diseases, such as periventricular leukomalacia, chronic lung disease, and bronchopulmonary dysplasia [4]. However, neonates are especially prone to oxidative stress, because they are often exposed to high oxygen concentrations due to the rapid passage from the intrauterine to the extrauterine environment, and they have limited antioxidant defense [4]. Therefore, improving antioxidant capacity in newborns is crucial.

Maternal nutrition during pregnancy causes permanent adaptations in the offspring, which probably occur because of epigenetic regulation and changes in metabolic programming [5]. The composition of diets in gestation has been reported to modulate maternal intestinal adaptations to pregnancy, affect placental function, and impair fetal gut development and immune status [6,7]. Therefore, the antioxidant capacity of offspring can be improved by regulating maternal nutrition. Increasing maternal fiber intake is an effective means to improve the antioxidant capacity of offspring [2]. Wang et al. [8] also reported that increasing maternal fiber intake during pregnancy enhances the antioxidative capacity of mothers and their offspring through increasing the total superoxide dismutase (SOD) and glutathione peroxidase (GSH-Px) activity and decreasing the serum malondialdehyde (MDA) concentration. The gut microbiota plays an important role in regulating human health [9]. A previous study reported that diet dominates host genotype in influencing the gut microbiota [10], and changes in dietary components can quickly lead to alterations in the composition of the microbiota [11]. The maternal gut microbiota drives early postnatal innate immune development [12]. Moreover, maternal fiber intake during gestation alters the intestinal microbiota in offspring [13], and the gut microbiota in vaginally delivered infants resembles that of their mothers [14]. Research has shown that the gut microbiota inhibits the NF-κB pathway, thus leading to the production of inflammatory cytokines and chemokines (tumor necrosis factor (TNF-α), interleukin-6 (IL-6), and MCP1), and suppressing the inflammatory response [15]. The inflammatory response is often accompanied by changes in oxidative stress, which are strongly linked to alterations in the gut microbiota [16,17]. HO-1, an important antioxidative enzyme regulating the ROS levels of cells, can be induced by enteric microbiota [18,19]. However, whether dietary fiber in gestation can change the composition of the gut microbiota and subsequently improve the antioxidative capacity and immune status of offspring by altering the maternal gut microbiota has not been reported.

Generally, soluble fibers (SFs) are fermented more than insoluble fibers (ISFs), but both trigger specific alterations in the composition and predicted functions of colonic bacterial communities [20]. Whether bacteria exist that specifically utilize SF and ISF, and how single or mixed types of dietary fiber in gestation might influence enteric microbial functions, host health, and metabolism, still remain unclear. In the present study, inulin (a typical fermentable SF) and natural cellulose (a typical ISF) [21], were selected as supplementary dietary fibers in a pregnant sow model, which is usually used as an animal model for humans to estimate diet–microbiota–health interactions [22].

## 2. Results

### 2.1. Changes in Antioxidant Parameters in Sow Plasma and Piglet Plasma and Liver

The effects of dietary fiber composition in pregnant sow diets on antioxidant parameters in sow and piglet plasma are shown in Table 1. The activity of catalase (CAT) in sows was significantly higher in R1 and R2 than in R3 and R4 (*p* < 0.05), and in R3 than in R4 (*p* < 0.05). The MDA concentrations in sows were dramatically lower in R1 and R2 than in R3 and R4 (*p* < 0.05). The total antioxidant capacity (T-AOC) in the plasma in piglets was significantly higher in R1 and R2 than in R4 (*p* < 0.05). Piglets in R1 and R2 had the highest plasma activity of GSH-Px, at levels significantly higher than those in R3 and R4 (*p* < 0.05). 

Moreover, the liver CAT activity in piglets (Table 2) was significantly higher in R1 and R2 than in R4 (*p* < 0.05); the activity of GSH-Px in R1 was higher than that in R3 and R4 at birth (*p* < 0.05), and newborn piglets showed higher GSH-Px activity in R2 than in R3 (*p* < 0.05).

### 2.2. Changes in Inflammatory Factors in Sow and Piglet Plasma

As shown in Table 3, sows in R1 and R2 had significantly lower plasma interleukin-6 (IL-6) levels than those in R3 and R4 (*p* < 0.05). In addition, the plasma tumor necrosis factor (TNF-α) levels in piglets were significantly higher in R3 than in R1 and R2 (*p* < 0.05), and in R4 than R1 (*p* < 0.05). The ISF/SF showed a trend toward affecting plasma interleukin-2 (IL-2) levels in neonatal piglets (*p* < 0.10). 

### 2.3. Changes in Relative mRNA Expression in Piglet Liver

**Figure 1:**
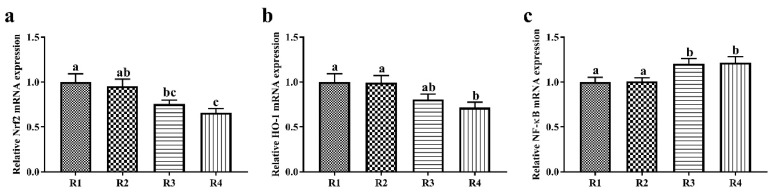
Effects of dietary fiber composition in pregnant sow diets on the relative expression of mRNA. (**a**) Nrf2; (**b**) HO-1; (**c**) NF-κB. R1, R2, R3, and R4 were diets in which the ratios of insoluble to soluble fiber were 3.89, 5.59, 9.12, and 12.81, respectively. Values are mean ± standard error (*n* = 6). ^a–c^ Means with different lowercase letters differ significantly among treatments (*p* < 0.05).

The effects of dietary fiber composition in pregnant sow diets on relative mRNA expression in the piglet liver are shown in Figure 1. The relative liver Nrf2 mRNA expression in piglets was significantly higher in R1 than in R3 and R4 (*p* < 0.05), and in R2 than in R4 (*p* < 0.05). Piglets in R1 and R2 had significantly higher relative HO-1 mRNA expression in the liver than piglets in R4 (*p* < 0.05). Moreover, the offspring of sows fed R1 and R2 diets showed significant downregulation of the relative liver NF-κB mRNA expression (*p* < 0.05).

### 2.4. Changes in Microbial Metabolites SCFAs in Sow Feces and Piglet Colonic Contents

The effects of dietary fiber composition in pregnant sow diets on short-chain fatty acids (SCFAs) production in piglet colonic contents are shown in Table 4. In sow feces, the acetate concentration of R1 was significantly higher than that in R3 and R4 (*p* < 0.05), and that in R2 was significantly higher than that in R4 (*p* < 0.05); the propionate concentration in R1 and R2 was significantly higher than that in R4 (*p* < 0.05); the total SCFAs concentration in R1 and R2 was higher than that in R4 (*p* < 0.05). In the piglet colonic contents, the acetate, butyrate, and total SCFAs concentrations in R1 and R2 were all higher than those in R3 and R4 (*p* < 0.05); the butyrate concentration in R1 and R2 was significantly higher than that in R4 (*p* < 0.05).

### 2.5. Changes in Microbial Composition and Diversity in Sow Feces

#### 2.5.1. Changes in Fecal Microbial Diversity

**Figure 2:**
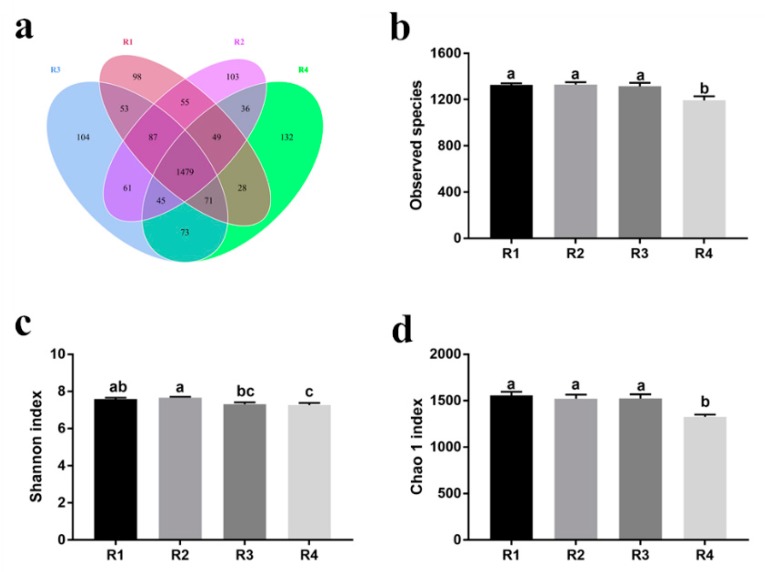
Comparison of the operational taxonomic units (OTUs) and alpha diversity analyses among treatments on day 110 of gestation. The observed OTUs share ≥97% sequence similarity. (**a**) A Venn diagram was generated to depict the common and unique OTUs among all treatments at day 110 of pregnancy. (**b**) The observed species, (**c**) Shannon index, and (**d**) Chao 1 index were used to ascertain differences in alpha diversity according to different diets. Sows were regarded as the experimental units (*n* = 6 per treatment). R1, R2, R3, and R4 were diets in which the ratios of insoluble to soluble fiber were 3.89, 5.59, 9.12, and 12.81, respectively. Values are mean ± standard error (*n* = 6). ^a–c^ Means with different superscripts within a row differ (*p* < 0.05).

Pairs comprising groups R2 and R4, and R1 and R4, shared fewer common operational taxonomic units (OTUs) with each other (Figure 2a). The number of observed species in R4 was significantly lower than that in the other treatments (*p* < 0.05) (Figure 2b). To assess fecal microbial community structure, we used the Shannon index and Chao 1 index (Figure 2c,d). The Shannon index was significantly higher in R1 than R4, and significantly higher in R2 than R3 and R4 (*p* < 0.05). Moreover, the Chao 1 index in R1, R2, and R3 was notably higher than that in R4 (*p* < 0.05).

**Figure 3:**
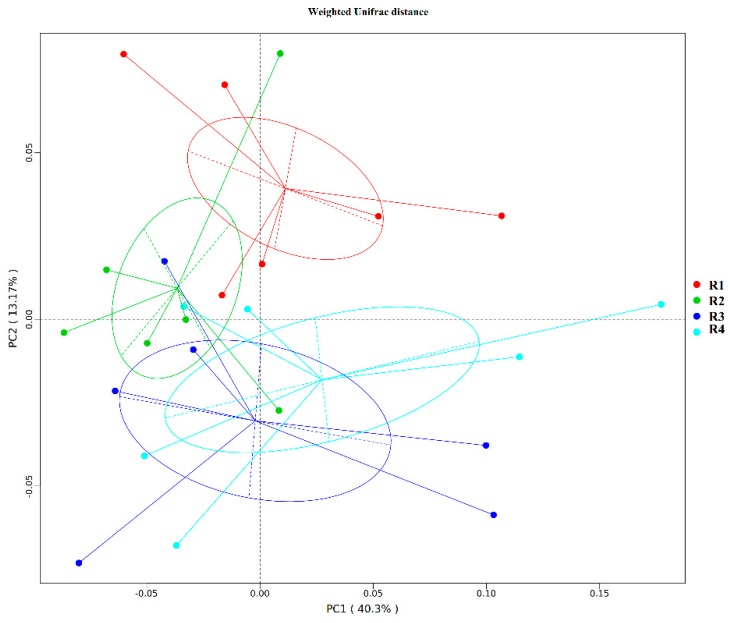
PCoA profile based on the weighted Unifrac distance of the OTUs in each sample on day 110 of pregnancy. R1, R2, R3, and R4 were diets in which the ratios of insoluble to soluble fiber were 3.89, 5.59, 9.12, and 12.81, respectively. *n* = 6 for all treatments.

As shown in Figure 3, there were different microbial community structures in fecal samples from after four treatments on day 110 of pregnancy. Analysis of similarities (Anosim) (Table S1) showed that R3 and R4 sows on day 110 of pregnancy had significantly different microbiota community structures than that in R1 sows (*p* < 0.05), and significantly different microbiota community structures also were found between R2 and R3 sows (*p* < 0.05).

#### 2.5.2. Changes in Relative Abundance at the Phylum Level

The relative abundance at the phylum level of sow fecal microbiota (top ten; Table 5) suggested that *Firmicutes* and *Bacteroidetes* were predominant. The relative abundance of bacteria from the phyla *Spirochaetes*, *Proteobacteria*, *Tenericutes*, and *Actinobacteria* significantly differed among groups (*p* < 0.05). The relative abundance of *Spirochaetes* in R2 and R3 was significantly higher than that in R4 (*p* < 0.05); *Proteobacteria* in R1 and R3 were signally lower than those in R4 (*p* < 0.05); *Actinobacteria* in R3 and R4 were significantly lower than those in in R2 (*p* < 0.05). The relative abundance of *Tenericutes* in R1 was significantly higher than that in R4 (*p* < 0.05).

#### 2.5.3. Changes in Relative Abundance at the Genus Level

**Figure 4:**
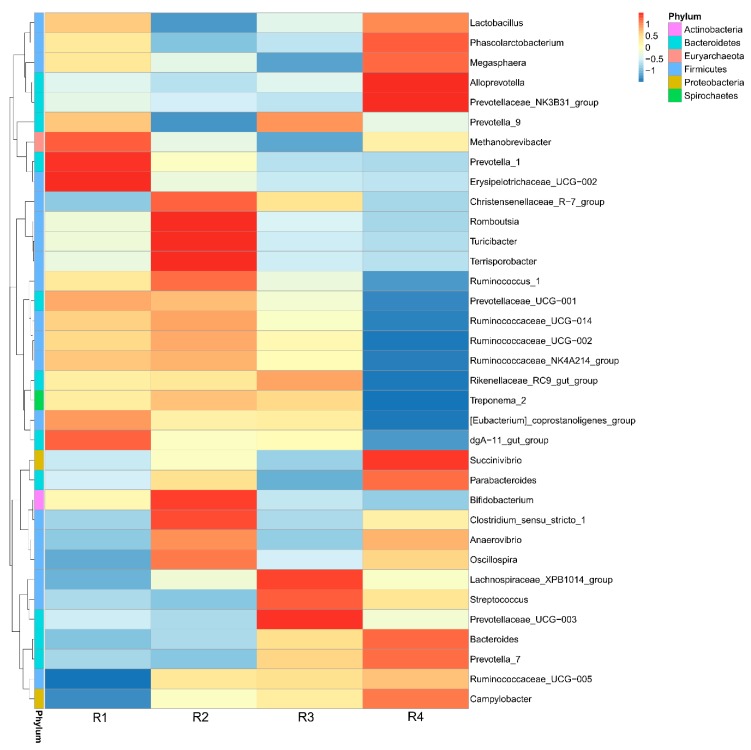
Heat map of the relative abundance of genera in sow feces on day 110 of pregnancy. Each vertical lane corresponds to one treatment. Different colors indicate the relative abundance of genera. R1, R2, R3, and R4 were diets in which the ratios of insoluble to soluble fiber were 3.89, 5.59, 9.12, and 12.81, respectively. *n* = 6 for all treatments.

The relative abundance at the genus level of sow fecal microbiota (top 35) is presented in Table S2 and shown in a heat map (Figure 4). *Streptococcus* and *Clostridium_sensu_stricto_1* were the top two genera. The relative abundance of *Streptococcus* in R3 and R4 was significantly higher than that in R1 and R2 (*p* < 0.05). *Treponema_2* in R2 and R3 were significantly higher than those in R4 (*p* < 0.05). *Ruminococcaceae_UCG-005* in R2, R3, and R4 were notably higher than those in R1 (*p* < 0.05). *Prevotella_1* and *Erysipelotrichaceae_UCG-002* in R3 and R4 were significantly lower than those in R1 (*p* < 0.05). *Bifidobacterium* in R3 and R4 were significantly lower than those in R2 (*p* < 0.05). *Ruminococcus_1* in R1 and R2 were significantly higher than those in R4 (*p* < 0.05). *Ruminococcaceae_UCG-014* in R1, R2, and R3 were significantly higher than those in R4 (*p* < 0.05). *Bacteroides* in R1 and R2 were clearly lower than those in R4 (*p* < 0.05). Levels of *[Eubacterium]_coprostanoligenes_group* and *Campylobacter* in R1 were significantly different from those in R4 (*p* < 0.05).

### 2.6. Changes in Microbial Composition and Diversity in Piglet Colonic Contents

#### 2.6.1. Changes in Colonic Microbial Diversity

**Figure 5:**
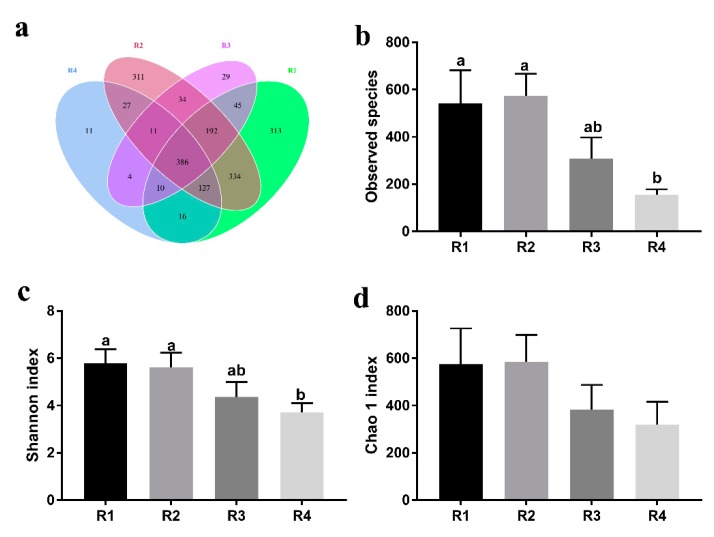
Comparison of the OTUs and alpha diversity analyses of piglet fecal microbiota among treatments. The observed OTUs share ≥97% sequence similarity. (**a**) A Venn diagram was generated to describe the common and unique OTUs among all treatments. (**b**) The observed species, (**c**) Shannon index, and (**d**) Chao 1 index were used to ascertain differences in alpha diversity according to different diets. Individual piglets were regarded as the experimental units (*n* = 6 per treatment). R1, R2, R3, and R4 were diets in which the ratios of insoluble to soluble fiber were 3.89, 5.59, 9.12, and 12.81, respectively. Values are mean ± standard error (*n* = 6). ^a–b^ Means with different superscripts within a row differ (*p* < 0.05).

R1 and R2 shared the most common OTUs (1039), whereas R3 and R4 had the fewest common OTUs (411) (Figure 5a). The observed species (Figure 5b) and Shannon indexes (Figure 5c) in R1 and R2 were all significantly higher than those in R4 (*p* < 0.05). No significant difference was observed in the Chao 1 index (*p* > 0.05, Figure 5d).

**Figure 6:**
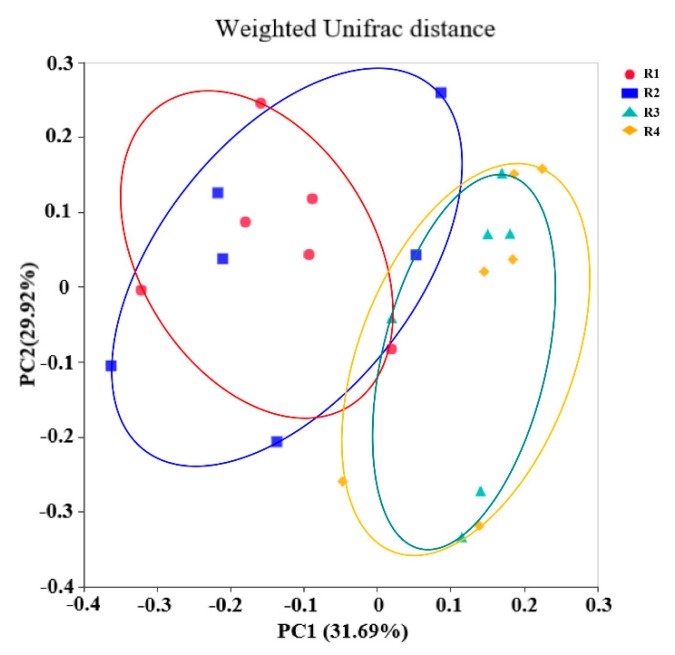
PCoA profile based on the weighted Unifrac distance of the OTUs in each neonatal piglet colon content sample. R1, R2, R3, and R4 were diets in which the ratios of insoluble to soluble fiber were 3.89, 5.59, 9.12, and 12.81, respectively. *n* = 6 for all treatments.

In addition, the PCoA profile for piglet colonic samples based on weighted Unifrac distance (Figure 6) showed a clear separation between group R1 and R2 and group R3 and R4 pairs. Anosim (Table S3) showed that R3 and R4 had significantly different microbiota community structures than R1 and R2 (*p* < 0.05); the microbiota structure in R1 was similar to that in R2, whereas that in R3 was similar to that in R4 (*p* > 0.05).

#### 2.6.2. Changes in Relative Abundance at the Phylum Level

**Figure 7:**
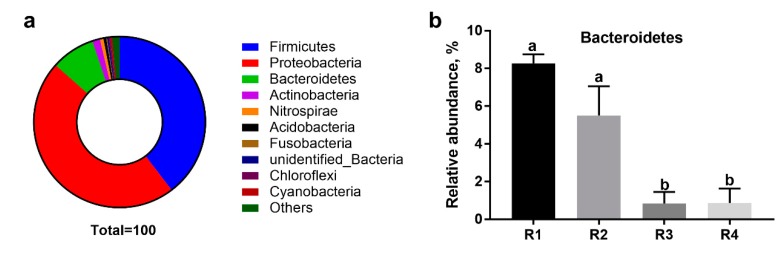
Effects of dietary fiber composition in sow gestation diets on the relative abundance of piglet colonic microbiota in phyla. (**a**) Circular diagram of the relative abundance of microbiota; (**b**) relative abundance of Bacteroidetes in four treatments. R1, R2, R3, and R4 were diets in which the ratios of insoluble to soluble fiber were 3.89, 5.59, 9.12, and 12.81, respectively. Values are mean ± standard error (*n* = 6). ^a,b^ Means with different lowercase letters differ significantly among treatments (*p* < 0.05).

The relative abundance at the phylum level in piglet colonic microbiota (top ten) is shown in Figure 7. Firmicutes, Proteobacteria, Bacteroidetes, and Actinobacteria were the most predominant phyla. Among these predominant phyla, the relative abundance of Bacteroidetes in R1 and R2 was significantly higher than in R3 and R4 (*p* < 0.05).

#### 2.6.3. Changes in Relative Abundance at the Genus Level

**Figure 8:**
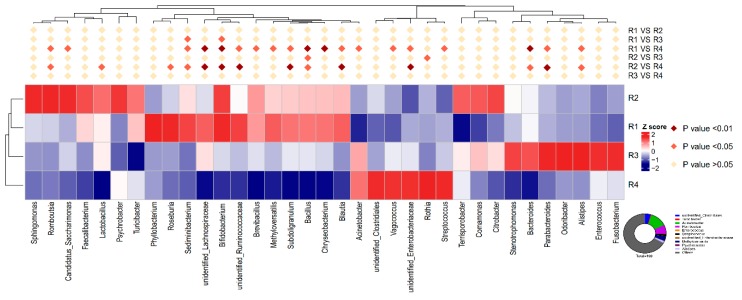
Heat map distribution of OTUs in piglet colonic contents for all treatments. Circular diagram represents the species with relative abundance in the top ten. The heat map shows the genera with relative abundance in the top 35. R1, R2, R3, and R4 were diets in which the ratios of insoluble to soluble fiber were 3.89, 5.59, 9.12, and 12.81, respectively. Different colors indicate the relative abundance of taxa. *n* = 6 for each treatment.

The relative abundance at the genus level in piglet colonic microbiota (top 35) is shown in Figure 8. *Acinetobacter* and *Romboutsia* were the top two genera. The relative abundance of *Romboutsia*, *Sediminibacterium*, *Bifidobacterium*, *unidentified_Lachnospiraceae*, *unidentified_Ruminococcaceae*, *Subdoligranulum*, *Bacillus*, *Blautia*, *Bacteroides,* and *ParaBacteroides* were all significantly higher in R1 and R2 than in R4 (*p* < 0.05). In addition, the relative abundance of *Sediminibacterium* and *Bifidobacterium* in R1 was notably higher than that in R3 (*p* < 0.05), and R2 showed a significantly higher relative abundance of Bacillus than R3 (*p* < 0.05). The relative abundance of *unidentified_Enterobacteriaceae* was significantly lower in R1 and R2 than in R4 (*p* < 0.05). Compared with R4, R1 had a significantly higher abundance of *Candidatus_Saccharimonas*, *Brevibacillus*, *Methyloversatilis,* and *Chryseobacterium* (*p* < 0.05), and a lower abundance of *Acinetobacter*, *Vagococcus,* and *Streptococcus* (*p* < 0.05).

## 3. Discussion

Newborns are vulnerable to free radical oxidative damage, thus resulting in oxidative stress [23], because neonates (i) are often exposed to high oxygen concentrations, (ii) have diminished antioxidant activity, and (iii) have infections or inflammation due to environmental microbiota. Oxidative stress probably contributes to the severity of several newborn conditions to an extent that may cause organ injury or even death [24]. Oxidative stress decreases the average daily feed intake, average daily gain, and nutrient digestibility in a pig model [25]. Pregnant women usually show increased oxidative damage during the third trimester of pregnancy [26]. Our previous study has indicated that maternal oxidative stress status might be transmitted to offspring by affecting placental oxidative stress, and improvements in maternal antioxidant capacity benefit fetal and neonatal development and health [27]. Thus, improving maternal oxidative stress status, which can be regulated by nutritional means, provides a favorable means of enhancing the antioxidant capacity of offspring. 

Dietary fiber is considered a key component in a healthful diet in pregnant women [28]. Pregnant rats fed a high-fiber diet (oat bran and wheat bran, 1:1, *w*/*w*, 250 g/kg diet) showed a higher antioxidative capacity than those fed a high-fat diet, and this response was also present in future generations, as represented by higher liver total SOD, and Cu- and Zn-containing SOD activity [2]. A similar study also shoed that a gestation diet supplemented with inulin enhances the serum activity of total SOD and GSH-Px and decreases the concentration of MDA in mothers and their offspring [6]. Complex enzymatic and nonenzymatic systems play vital roles in protecting organisms from oxidative damage [29]. In the present study, sows fed R1 and R2 diets showed increased CAT activity in sow plasma, and the GSH-Px activity increased in piglet plasma. SOD, CAT, and GSH-Px are three crucial endogenous antioxidant enzymes that play important roles in preventing oxidative damage. SOD converts ROS into hydrogen peroxide (H_2_O_2_), and then CAT and GSH-Px degrade the H_2_O_2_ to water and oxygen [30,31]. In addition, sows fed R1 and R2 diets showed decreased plasma MDA concentrations, and the piglets showed increased T-AOC in our study. T-AOC and MDA are two non-enzymatic indicators of antioxidant status and cell damage, respectively. In detail, T-AOC is an important integrative index reflecting the total antioxidant capacity of the body [32], whereas MDA is a secondary product of lipid oxidation and is closely associated with cell damage, for which MDA has been widely considered an index to monitor the degree of lipid peroxidation [33]. The liver, an important metabolic organ, plays a crucial role in nutrient metabolism and transformation, and in the defense against the invasion of bacteria and bacterial products [34]. We also found that the offspring of sows fed R1 and R2 diets showed increased liver CAT and GSH-Px activity, and elevated mRNA expression of Nrf2 and HO-1 in the liver. Nrf2, a key transcription factor, plays an essential role in regulating the activity of endogenous antioxidant enzymes to resist oxidative stress [35]. A previous study in piglets also showed that the activity of antioxidative enzymes is enhanced by increasing the mRNA expression level of Nrf2 [36]. Moreover, Nrf2 regulates the activity of HO-1, an important antioxidative enzyme regulating the ROS levels of cells [19] and serving as a sensitive and reliable indicator of cellular oxidative stress [37]. Therefore, R1 and R2 diets enhanced the antioxidative capacity of offspring through upregulating Nrf2 and HO-1 mRNA expression and improving the antioxidant enzyme activity.

Oxidative stress is often associated with the inflammatory response. Cytokines have crucial roles in the immune and inflammatory response [38,39]. In the present study, we found that sows fed R1 and R2 had lower plasma IL-6, and piglets showed lower plasma TNF-α. TNF-α and IL-6 are both pro-inflammatory cytokines. IL-6 is a pleiotropic cytokine participating in the physiology of virtually every organ system, and it activates the hypothalamic-pituitary-adrenal axis and regulates hepatic protein synthesis during the acute response [40]. In addition, IL-6 plays an important role in regulating the balance between the IL-17-producing Th17 cells and regulatory T cells, which have prominent roles in immune functions [41]. TNF-α is secreted by activated macrophages and has some metabolic effects on lipid metabolism [42]. TNF-α signaling induces activation of the transcription factor NF-κB and programmed cell death [43]. The NF-κB signaling pathway is considered a key inducer of inflammation. Accordingly, lower mRNA expression levels of NF-κB were observed in the liver in R1 and R2 piglets. Similar research has shown that a maternal SF diet increases the plasma concentrations of anti-inflammatory factors, such as interleukin 10 (IL-10) and transforming growth factor β, in offspring [44]. Therefore, the R1 and R2 diets decreased the inflammatory response of offspring through decreasing the plasma TNF-α level and liver NF-κB mRNA expression. Many studies have shown that high dietary fiber intake is associated with decreased inflammation [45,46]. Accumulating evidence indicates that maternal inflammation also has long-term consequences for offspring by affecting the intrauterine environment [47]. In our current study, R1 and R2 diets improved the antioxidative capacity and decreased the plasma pro-inflammatory cytokine concentration in sows and piglets. Continued oxidative stress leads to chronic inflammation [48] and detrimentally affects growth performance [49]. Our previous study showed increased average daily gain of piglets during lactation when the ISF/SF ratios in the pregnancy diet were 3.89 and 5.59 [50]. Therefore, the dietary fiber composition in the maternal pregnancy diet has an important effect on the health of mothers and offspring, and there is a threshold ratio of insoluble to soluble fiber to ensure that the dietary fiber is effective. 

The gut microbiota has an indispensable role in host health by promoting the development of the immune system, decreasing inflammation, and competitively inhibiting pathogens [51]. Consumption of dietary fiber is an effective strategy for modulating the microbiota [52]. The composition of the gut microbiota is also affected by the types of dietary fiber [20]. In the current study, we found that sows in R1 and R2 on day 110 of pregnancy had significantly different microbiota community structures than those in R3 and R4, and R1 and R2 diets increased the α-diversity indexes of the sow fecal microbiota, which also indicated a threshold ratio of insoluble to soluble fiber. Low microbial diversity is often associated with metabolic syndrome and inflammation [53]. Moreover, the R1 and R2 diets decreased the abundance of *Streptococcus* and increased the abundance of *Bifidobacterium* in sow feces. A higher abundance of *Streptococcus* is related to numerous inflammatory responses [54], whereas *Bifidobacterium* decreases inflammation through inhibiting the growth of pathogens via the production of organic acids and releasing soluble factors that alleviate the secretion of pro-inflammatory cytokines by immune cells [55,56]. The gut microbiota of individuals is dominated by different fiber-utilizing bacteria, which ferment dietary fiber into SCFAs, including acetate, propionate, and butyrate, which are important for human health. SCFAs, mainly butyrate, suppress the LPS- and cytokine-stimulated production of pro-inflammatory mediators, including TNF-α and IL-6, via interaction with the orphan G protein-coupled receptors GPR41 and GPR43 [57,58]. In the current study, sows in R1 and R2 had higher fecal SCFA concentrations, which might have resulted from soluble fiber being more easily fermented to produce SCFAs than ISF. In addition, SCFAs produced by the intestinal microbiota or their specific GPR43 agonist have been reported to inhibit oxidative stress [59]. Interestingly, piglets in R1 and R2 also showed increased SCFA concentrations in the colon. SCFAs, especially butyrate, produced by microbial fermentation in the colon, were reported to raise Nrf2 in colonocytes [60] and modulate the activity of the transcription factor NF-κB [61], which might be the reason for the decreased inflammatory response and increased antioxidative capacity. Different SCFA concentrations are related to the distinct compositions of the microbiota among the four treatments. The offspring of sows fed R1 and R2 diets showed increased abundance of degrading bacteria, such as *Bacteroidetes*, *Romboutsia*, *Ruminococcaceae,* and *Parabacteroides*, in the colonic contents. Recently, sequencing has indicated that placenta and umbilical cord blood is not sterile, and the microbiota within the neonate’s meconium shares significant similarity with that of the placenta, thus suggesting that maternal transfer of microbiota is possible and might occur during gestation [62,63,64]. Furthermore, previous research has also shown that vaginally delivered infants acquire bacterial communities resembling their own mother’s vaginal microbiota [65]. Therefore, the maternal microbiota plays a vital role in the composition of the intestinal flora of the offspring. Pregnancy-related changes in the maternal microbiota are dependent on the mother’s periconceptional diet [66]. Therefore, diet composition during pregnancy has important effects on the structure of the gut microbiota of offspring [67]. Paßlack et al. [13] showed that the addition of inulin to a gestation diet modulates not only the intestinal microbiota in sows but also their offspring. In the current study, similar structures of microbiota were also observed between mothers and their offspring: R1 resembled R2, and R3 resembled R4, and the observed changes in the relative abundance of *Streptococcus* and *Bifidobacterium* in the offspring was similar to that in the mother. Moreover, decreased abundance of *Enterobacteriaceae* was also observed in the colonic contents of piglets in R1 and R2, in agreement with the results for inflammatory factors. The capacity of *Enterobacteriaceae* to induce host inflammation through endotoxin production is well known [68]. Increased *Bifidobacterium* inhibits the overgrowth of *Enterobacteriaceae* [69]. Moreover, the relative abundance of *Acinetobacter* in the colonic contents of R1 was lower than that of R4, but not significantly different from that of R2. *Acinetobacter*, a highly concerning pathogen belonging to the gram-negative *Coccobacillus*, has become an increasingly common nosocomial problem [70]. Members of the genus *Acinetobacter* have been implicated in a wide spectrum of infectious diseases [71] and have a strong ability to acquire or upregulate antibiotic drug resistance determinants [72]. These findings might also suggest that the offspring of sows in R1 and R2 had a strong ability to resist *Acinetobacter* infection. The increased beneficial bacteria and decreased harmful bacteria might be another reason for the lower inflammation and oxidative stress in R1 and R2 piglets. 

Thus, the composition of dietary fiber in the pregnancy diet has an important role in improving antioxidative capacity and decreasing the inflammatory response of mothers and their offspring through modulating the composition of the gut microbiota. Not only the dietary fiber level but also the ratio of insoluble dietary fiber to soluble dietary fiber should be considered in pregnancy diets.

## 4. Materials and Methods 

### 4.1. Ethical Approval

The present experiment was conducted at the Research Farm of Animal Nutrition Institute, Sichuan Agricultural University, Ya’an, China. The experimental protocol used in the present study was approved by the Animal Care and Use Committee of Sichuan Agricultural University and followed the current laws regarding animal protection (Ethics Approval Code: SCAUAC201408-3; date: 15 August 2016).

### 4.2. Animals and Diets

A total of 64 Large White × Landrace crossbred gilts with similar body weight (BW) and backfat (BF) thickness were used in this study. After artificial insemination, gilts were assigned randomly to 4 treatments (16 replicates per treatment) and fed diets with the same level of dietary fiber but different ratios of ISF to SF of 3.89, 5.59, 9.12, and 12.81, denoted R1, R2, R3 and R4, respectively. All sows were fed the same amount of feed during the entire gestation. In detail, sows were fed 2.37 kg/d of corresponding diet from days 1 to 90 of gestation, 2.86 kg/d diet from days 91 to 112 of gestation, and 1.90 kg/d diet from day 113 to parturition. Sows were fed once per day at 9:00. Sows were moved from gestation to farrowing rooms on day 110 of gestation and were kept in individual farrowing crates thereafter. The ingredients and nutrient composition of the gestation diets are shown in Table S4. All diets based on corn-soybean meal were formulated to meet or exceed the nutrient requirements of gestating sows, as recommended by the NRC (2012), and to contain the same content of all nutrients except SF and ISF, whose ratios were adjusted. The inulin used in the study was obtained from ZTH Tech (Beijing, China), and cellulose was obtained from Guangxi Shangda Tech Co. (Guangxi, China). The purity of inulin and cellulose was >90%.

### 4.3. Sampling Procedure

Fasting blood samples (10 mL) were collected from the ear veins of eight healthy sows on d 110 of gestation in the morning. Blood samples were collected into two tubes containing heparin sodium and allowed to stand at room temperature for 30 min, then centrifuged for 15 min at 3000× *g* at 4 °C. Plasma samples were harvested and stored at −20 °C until analysis. Meanwhile, fresh fecal samples (about 2 g) from six healthy sows were collected from the rectum before feeding in the morning; the samples were divided into two sterile tubes for the detection of SCFAs and analysis of microbiota, and then stored at −80 °C immediately until analysis. 

A total of 24 piglets (six per treatment: three males and three females) that had not eaten clostrum from different sows, with BWs closest to the average, were slaughtered immediately after birth for sampling after deep anesthesia with Zoletile 50 (Virbac, SA, Carros, France) administered by intramuscular injection at a dose of 0.1 mg/kg of body weight. Approximately 10 mL of blood per pig was drawn by venipuncture in the jugular vein. The plasma was obtained as described above. After evisceration, the colon contents were collected on a clean bench, immediately placed in sterile bags and stored at –80 °C for microbiological analysis. In addition, approximately 0.5 g liver samples from the same location were obtained and stored at −80 °C until analysis.

### 4.4. Analysis of Oxidative and Antioxidative Parameters

The oxidative and antioxidative parameters in the blood and liver, including T-AOC, CAT, total SOD, GSH-Px, and MDA, were measured with specific assay kits (Nanjing Jiancheng Bioengineering Institute, Nanjing, China). All variables in the blood and liver samples were measured according to the protocol from a previous study [6].

### 4.5. Analysis of Inflammatory Factors

Plasma IL-2, IL-6, IL-10, and TNF-α concentrations of sows and piglets were determined with commercial ELISA kits (Feiya Biotechnology Co. Ltd., Yancheng, China).

### 4.6. Measurement of Gene Expression

A quantitative real-time polymerase chain reaction (PCR) detection system (Bio-Rad Laboratories, CA, USA) was used to analyze mRNA expression levels of Nrf2, HO-1, and NF-κB in piglet liver samples. Frozen liver tissue samples (50–100 mg) were ground to powder with a mortar while liquid nitrogen was added continously. Total RNA was extracted with TRIzol reagent (Invitrogen, Carlsbad, CA, USA), and this was followed by RNA quality determination. The absorbance of RNA solution at wavelengths of 260 and 280 nm was detected with a scanning spectrophotometer (Beckman DU-800, Beckman Coulter Inc., Brea, CA, USA), and RNA concentrations were confirmed with a nucleic-acid/protein analyzer (Beckman DU-800, Beckman Coulter Inc., Brea, CA, USA). Then cDNA was synthesized with a commercial reverse transcription (RT) kit (TaKaRa Biotechnology, Shiga, Japan) according to the manufacturer’s instructions and stored at −20 °C for relative quantification by PCR. Primer sequences (Table S5) used for real-time PCR were as described by Chen et al. [36]. cDNA was amplified with an ABI 7900HT instrument (Applied Biosystems, Foster City, CA, USA). The mixture (10 μL) contained 5 μL of SYBR Green Supermix (TaKaRa, Shiga, Japan), 1 μL of cDNA template, 0.4 μL of each primer (10 mM), 0.2 μL of ROX Reference Dye, and 3 μL doubled-distilled water. The cycling conditions were as follows: pre-denaturation at 95 °C for 30 s, followed by 40 cycles of denaturation at 95 °C for 10 s, and annealing at 60 °C for 25 s. After amplification, melting curve analysis (50–95 °C with a heating rate of 0.1 °C/s and a continuous fluorescence measurement) was performed. β-actin, as an internal control, was amplified in parallel with the target gene to allow for gene normalization and quantification. All samples were measured in triplicate, and the product size was determined by agarose gel electrophoresis. The relative mRNA abundance of the genes detected in the liver samples was calculated with the 2^−ΔΔCt^ method.

### 4.7. Detection of Fecal SCFAs

The SCFAs in sow feces and piglet colonic contents were measured through a gas chromatographic method as previously described in Zhou et al. [73]. Briefly, 0.7 g fecal samples were suspended in 1.5 mL of distilled water and allowed to stand for 30 min, then centrifuged (15,000× *g*) at 4 °C for 15 min. Then 1 mL supernatant was transferred and mixed with 0.2 mL metaphosphoric acid (25%, *w*/*v*) and 23.3 µL crotonic acid (210 mmol/L, internal standard). After the samples were allowed to stand at 4 °C for 30 min, they were centrifuged (15,000× *g*) at 4 °C for 10 min again. Then 0.3 mL supernatant was transferred and mixed with 0.3 mL methanol. A small aliquot of the supernatant (1 µL) was analyzed with a gas chromatograph (Varian CP-3800 GC, Palo Alto, CA, USA).

### 4.8. Microbial Analysis

Bacterial genomic DNA was extracted from frozen sow fecal samples and neonatal piglet colonic contents with an E.Z.N.A. TM Stool DNA kit (Omega Bio-Tek, Norcross, GA, USA). Sequencing and data analysis were subsequently performed on the Illumina HiSeq PE250 platform by Novogene (Beijing, China), as previously described in Zhou et al. [73]. Sequences were clustered into the same OTUs at 97% sequence similarity. The alpha diversity and beta diversity were calculated for comparison of taxonomic data. The observed species, Chao 1 index, and Shannon index were used to determine differences in alpha diversity according to different diets. Unifrac weighted distance matrices were calculated, and analysis of similarities (Anosim) was used to access differences among the microbial communities. All analyses from clustering to alpha and beta diversity were performed in QIIME (V1.7.0) and displayed in R software (V2.15.3).

### 4.9. Statistical Analysis

Individual sow or piglet data were used to evaluate variables in PROC GLM of SAS (9.0 Inst. Inc., Cary, NC, USA). Variations among the four treatments were compared with Duncan’s multiple comparisons test. Statistical analyses for the relative abundance of the phyla and genera and the diversity indices and estimators were performed with one-way ANOVA in SAS (9.0 Inst. Inc., Cary, NC, USA). The normality of the data was assessed with the Shapiro–Wilk statistic (*w* > 0.05). If the data did not follow a normal distribution, transformation was used to achieve normality. Values are expressed as mean ± SEM in tables and figures. Statistical significance was set at *p* < 0.05.

## Figures and Tables

**Table 1:** Effects of dietary fiber composition in pregnant sow diets on antioxidant parameters in sow and piglet plasma.

Parameters ^2^	Treatments ^1^				*p*-Value
	R1	R2	R3	R4	
Sow plasma					
T-AOC, U/mL	1.16 ± 0.21	1.70 ± 0.14	1.90 ± 0.31	1.44 ± 0.30	0.202
CAT, U/mL	5.95 ± 0.37 ^a^	5.70 ± 0.24 ^a^	3.05 ± 0.19 ^b^	2.22 ± 0.22 ^c^	<0.001
T-SOD, U/mL	0.94 ± 0.02	0.95 ± 0.05	0.83 ± 0.03	0.86 ± 0.02	0.052
GSH-Px, U/mL	1012.42 ± 51.68	941.36 ± 80.10	946.68 ± 47.26	966.66 ± 31.76	0.860
MDA, mmol/mL	3.19 ± 0.30 ^bc^	2.57 ± 0.29 ^c^	3.54 ± 0.25 ^ab^	4.19 ± 0.24 ^a^	0.004
Piglet plasma					
T-AOC, U/mL	3.70 ± 0.15 ^a^	4.06 ± 0.53 ^a^	3.14 ± 0.52 ^ab^	2.22 ± 0.49 ^b^	0.035
CAT, U/mL	4.47 ± 0.35	5.47 ± 0.71	4.45 ± 0.12	3.55 ± 0.44	0.054
T-SOD, U/mL	0.27 ± 0.04	0.27 ± 0.05	0.25 ± 0.04	0.25 ± 0.04	0.977
GSH-Px, U/mL	266.16 ± 31.82 ^a^	223.66 ± 21.67 ^a^	112.25 ± 20.80 ^b^	104.49 ± 22.64 ^b^	<0.001
MDA, mmol/mL	4.72 ± 0.23	5.16 ± 0.60	5.41 ± 0.41	6.08 ± 0.96	0.440

^1^ R1, R2, R3, and R4 were diets in which the ratios of insoluble to soluble fiber were 3.89, 5.59, 9.12, and 12.81, respectively. ^2^ T-AOC, total antioxidant capacity; CAT, catalase; T-SOD, total peroxide dismutase; MDA, malondialdehyde; GSH-Px, glutathione peroxidase. Values are mean ± standard error (*n* = 8 for sows, *n* = 6 for piglets). ^a–c^ Means with different superscripts within a row differ (*p* < 0.05).

**Table 2:** Effects of dietary fiber composition in pregnant sow diets on antioxidant parameters in piglet livers.

Parameters ^2^	Treatments ^1^				*p*-Value
	R1	R2	R3	R4	
T-AOC, U/mL	2.38 ± 0.58	2.59 ± 0.52	2.16 ± 0.56	2.22 ± 0.30	0.924
CAT, U/mL	11.75 ± 0.94 ^a^	11.00 ± 0.90 ^a^	7.47 ± 1.03 ^ab^	3.55 ± 0.69 ^b^	0.041
T-SOD, U/mL	16.65 ± 2.18	17.64 ± 2.46	13.26 ± 1.64	10.35 ± 1.20	0.059
GSH-Px, U/mL	68.99 ± 12.39 ^a^	50.68 ± 7.20 ^ab^	14.21 ± 1.71 ^c^	32.71 ± 5.56 ^bc^	0.001
MDA, mmol/mL	1.22 ± 0.46	1.03 ± 0.19	1.76 ± 0.46	0.97 ± 0.22	0.404

^1^ R1, R2, R3, and R4 were diets in which the ratios of insoluble to soluble fiber were 3.89, 5.59, 9.12, and 12.81, respectively. ^2^ T-AOC, total antioxidant capacity; CAT, catalase; T-SOD, total peroxide dismutase; MDA, malondialdehyde; GSH-Px, glutathione peroxidase. Values are mean ± standard error (*n* = 6). ^a–c^ Means with different superscripts within a row differ (*p* < 0.05).

**Table 3:** Effects of dietary fiber composition in pregnant sow diets on inflammatory factors in sow and piglet plasma.

Items ^2^	Treatments ^1^				*p*-Value
	R1	R2	R3	R4	
Sow plasma					
IL-2, pg/mL	479.67 ± 39.03	527.00 ± 47.89	544.80 ± 34.18	458.03 ± 29.60	0.423
IL-6, ng/L	942.65 ± 51.78 ^b^	919.20 ± 52.88 ^b^	1152.62 ± 62.17 ^a^	1128.28 ± 52.20 ^a^	0.012
IL-10, ng/L	177.30 ± 10.13	191.15 ± 14.68	170.81 ± 9.18	198.84 ± 14.94	0.458
TNF-α, pg/mL	389.76 ± 34.64	435.95 ± 31.29	405.95 ± 40.61	350.33 ± 20.91	0.303
Piglet plasma					
IL-2, pg/mL	542.91 ± 38.96	581.53 ± 34.17	493.44 ± 23.85	464.82 ± 18.47	0.068
IL-6, ng/L	968.27 ± 109.26	1013.16 ± 96.44	1087.09 ± 68.82	1030.72 ± 95.86	0.827
IL-10, ng/L	177.49 ± 11.23	185.24 ± 13.79	190.35 ± 14.62	195.09 ± 14.42	0.830
TNF-α, pg/mL	310.51 ± 21.47 ^a^	392.80 ± 35.92 ^ab^	485.21 ± 10.60 ^c^	448.17 ± 32.04 ^bc^	0.002

^1^ R1, R2, R3, and R4 were diets in which the ratios of insoluble to soluble fiber were 3.89, 5.59, 9.12, and 12.81, respectively. ^2^ IL-2, interleukin-2; IL-6, interleukin-6; IL-10, interleukin-10; TNF-α, tumor necrosis factor-α. Values are mean ± standard error (*n* = 8 for sows, *n* = 6 for piglets). ^a–c^ Means with different superscripts within a row differ (*p* < 0.05).

**Table 4:** Effects of dietary fiber composition in pregnant sow diets on the production of short-chain fatty acids in sow feces and piglet colonic contents.

Parameters ^2^	Treatments ^1^				*p*-Value
	R1	R2	R3	R4	
Sow feces					
Acetate	47.17 ± 2.73 ^a^	42.56 ± 1.05 ^ab^	36.12 ± 2.54 ^bc^	31.97 ± 3.75 ^c^	0.005
Propionate	18.34 ± 1.45 ^a^	18.50 ± 1.22 ^a^	17.09 ± 1.60 ^ab^	12.87 ± 1.13 ^b^	0.048
Butyrate	9.01 ± 0.65	7.52 ± 0.48	8.75 ± 2.19	6.09 ± 1.35	0.425
Total SCFAs ^2^	74.52 ± 4.66 ^a^	68.59 ± 2.04 ^a^	61.96 ± 2.10 ^ab^	50.93 ± 6.59 ^b^	0.008
Piglet colonic contents					
Acetate	14.72 ± 1.62 ^a^	12.78 ± 0.97 ^a^	6.59 ± 1.09 ^b^	4.14 ± 1.23 ^b^	0.001
Propionate	0.62 ± 0.24	0.78 ± 0.20	0.30 ± 0.10	0.20 ± 0.02	0.082
Butyrate	1.76 ± 0.46 ^a^	1.60 ± 0.32 ^a^	0.59 ± 0.18 ^b^	0.64 ± 0.26 ^b^	0.031
Total SCFAs ^2^	17.10 ± 2.21 ^a^	15.15 ± 0.87 ^a^	7.47 ± 1.32 ^b^	4.99 ± 1.51 ^b^	0.001

^1^ R1, R2, R3, and R4 were diets in which the ratios of insoluble to soluble fiber were 3.89, 5.59, 9.12, and 12.81, respectively. ^2^ SCFAs, short-chain fatty acids. Values are mean ± standard error (*n* = 8 for sows, *n* = 6 for piglets). ^a–c^ Means with different superscripts within a row differ (*p* < 0.05).

**Table 5:** Effects of dietary fiber composition in pregnant sow diets on the relative abundance of sow fecal microbiota at the phylum level.

Taxonomy, %	Treatments ^1^				*p*-Value
	R1	R2	R3	R4	
*Firmicutes*	52.57 ± 2.07	55.87 ± 1.77	55.48 ± 2.21	54.83 ± 2.79	0.663
*Bacteroidetes*	33.06 ± 1.79	28.94 ± 1.69	32.42 ± 2.53	32.89 ± 2.32	0.368
*Spirochaetes*	4.50 ± 0.89 ^ab^	5.14 ± 0.65 ^a^	4.93 ± 0.92 ^a^	2.20 ± 0.63 ^b^	0.020
*Proteobacteria*	2.63 ± 0.39 ^b^	3.40 ± 0.46 ^ab^	2.40 ± 0.26 ^b^	4.48 ± 0.72 ^a^	0.009
*Tenericutes*	2.17 ± 0.17 ^a^	1.61 ± 0.13 ^ab^	1.42 ± 0.25 ^ab^	1.06 ± 0.23 ^b^	0.013
*Euryarchaeota*	2.37 ± 0.33	1.45 ± 0.34	0.98 ± 0.38	1.70 ± 0.33	0.095
*Actinobacteria*	1.30 ± 0.23 ^ab^	2.03 ± 0.66 ^a^	0.97 ± 0.09 ^b^	0.92 ± 0.08 ^b^	0.018
*Verrucomicrobia*	0.96 ± 0.23	0.75 ± 0.11	0.72 ± 0.06	0.54 ± 0.09	0.133
*Cyanobacteria*	0.35 ± 0.12	0.19 ± 0.03	0.21 ± 0.04	0.47 ± 0.23	0.106
*Planctomycetes*	0.13 ± 0.03	0.23 ± 0.08	0.19 ± 0.12	0.27 ± 0.14	0.567

^1^ R1, R2, R3, and R4 were diets in which the ratios of insoluble to soluble fiber were 3.89, 5.59, 9.12, and 12.81, respectively. Values are mean ± standard error (*n* = 8 for sows, *n* = 6 for piglets). ^a–c^ Means with different superscripts within a row differ (*p* < 0.05).

## Supplementary Materials

Supplementary materials can be found at https://www.mdpi.com/1422-0067/21/1/31/s1. Table S1: Analysis of similarities (Anosim) to determine the beta diversity of sow fecal microbiota on day 110 of pregnancy; Table S2: Effects of dietary fiber composition in gestation diets on the relative abundance of sow fecal microbiota at the genus level; Table S3: Analysis of similarities (Anosim) to determine the beta diversity of neonatal piglet colonic microbiota; Table S4: Compositions of the experimental diets during pregnancy (as-fed basis); Table S5: Primer sequences used for quantitative real-time PCR.

## Abbreviations

T-AOC	Total antioxidant capacity
CAT	Catalase
T-SOD	Total peroxide dismutase
GSH-Px	Glutathione peroxidase
MDA	Malondialdehyde
IL-2	Interleukin-2
IL-6	Interleukin-6
IL-10	Interleukin-10
TNF-α	Tumor necrosis factor-α
SCFAs	Short-chain fatty acids
OTUs	Operational taxonomic units

## References

[1] Nappi A.J., Vass E. (1998). Hydroxyl radical formation via iron-mediated Fenton chemistry is inhibited by methylated catechols. Biochim. Biophys. Acta General Subj..

[2] Lin Y., Han X.F., Fang Z.F., Che L.Q., Wu D., Wu C.M. (2012). The beneficial effect of fiber supplementation in high- or low-fat diets on fetal development and antioxidant defense capacity in the rat. Eur. J. Nutr..

[3] Rezaie A., Parker R.D., Abdollahi M. (2007). Oxidative stress and pathogenesis of inflammatory bowel disease, an epiphenomenon or the cause?. Dig. Dis. Sci..

[4] Mutinati M., Pantaleo M., Roncetti M., Piccinno M., Rizzo A., Sciorsci R. (2014). Oxidative stress in neonatology. A review. Reprod. Domest. Anim..

[5] Barker D. (1990). The fetal and infant origins of adult disease. BMJ.

[6] Gohir W., Kennedy K.M., Wallace J.G., Saoi M., Britz-McKibbin P., Petrik J.J., Surette M.G., Sloboda D.M. (2018). High-fat diet intake modulates maternal intestinal adaptations to pregnancy, and results in placental hypoxia and impaired fetal gut development. bioRxiv.

[7] Vuillermin P.J., Macia L., Nanan R., Tang M.L., Collier F., Brix S. (2017). The maternal microbiome during pregnancy and allergic disease in the offspring. Seminars in Immunopathology.

[8] Wang Y.S., Zhou P., Liu H., Li S., Zhao Y., Deng K., Cao D.D., Che L.Q., Fang Z.F., Xu S.Y. (2016). Effects of inulin supplementation in low- or high-fat diets on reproductive performance of sows and antioxidant defence capacity in sows and offspring. Reprod. Domest. Anim..

[9] Hooper L.V., Littman D.R., Macpherson A.J. (2012). Interactions between the microbiota and the immune system. Science.

[10] Carmody R.N., Gerber G.K., Luevano J.M., Gatti D.M., Somes L., Svenson K.L., Turnbaugh P.J. (2015). Diet dominates host genotype in shaping the murine gut microbiota. Cell Host Microbe.

[11] Flint H.J., Scott K.P., Duncan S.H., Louis P., Forano E. (2012). Microbial degradation of complex carbohydrates in the gut. Gut Microbes.

[12] de Agüero M.G., Ganal-Vonarburg S.C., Fuhrer T., Rupp S., Uchimura Y., Li H., Steinert A., Heikenwalder M., Hapfelmeier S., Sauer U. (2016). The maternal microbiota drives early postnatal innate immune development. Science.

[13] Paßlack N., Vahjen W., Zentek J. (2015). Dietary inulin affects the intestinal microbiota in sows and their suckling piglets. BMC Vet. Res..

[14] Bäckhed F., Roswall J., Peng Y., Feng Q., Jia H., Kovatcheva-Datchary P., Li Y., Xia Y., Xie H., Zhong H. (2015). Dynamics and stabilization of the human gut microbiome during the first year of life. Cell Host Microbe.

[15] Belkaid Y., Hand T.W. (2014). Role of the microbiota in immunity and inflammation. Cell.

[16] de Vega J.M.A., Díaz J., Serrano E., Carbonell L.F. (2002). Oxidative stress in critically ill patients with systemic inflammatory response syndrome. Crit. Care Med..

[17] Qiao Y., Sun J., Ding Y., Le G., Shi Y. (2013). Alterations of the gut microbiota in high-fat diet mice is strongly linked to oxidative stress. Appl. Microbiol. Biotechnol..

[18] Onyiah J.C., Sheikh S.Z., Maharshak N., Otterbein L.E., Plevy S.E. (2014). Heme oxygenase-1 and carbon monoxide regulate intestinal homeostasis and mucosal immune responses to the enteric microbiota. Gut Microbes.

[19] Bao L., Li J., Zha D., Zhang L., Gao P., Yao T., Wu X. (2018). Chlorogenic acid prevents diabetic nephropathy by inhibiting oxidative stress and inflammation through modulation of the Nrf2/HO-1 and NF-ĸB pathways. Int. Immunopharmacol..

[20] Luo Y., Zhang L., Li H., Smidt H., Wright A.D.G., Zhang K., Ding X., Zeng Q., Bai S., Wang J. (2017). Different types of dietary fibers trigger specific alterations in composition and predicted functions of colonic bacterial communities in BALB/c mice. Front. Microbiol..

[21] Jonathan M.C., Borne J.J.G.C.v.d., Wiechen P.V., Silva C.S.D., Scholsa H.A. (2012). In vitro fermentation of 12 dietary fibres by faecal inoculum from pigs and humans. Food Chem..

[22] Heinritz S.N., Mosenthin R., Weiss E. (2013). Use of pigs as a potential model for research into dietary modulation of the human gut microbiota. Nutr. Res. Rev..

[23] Saugstad O. (1996). Mechanisms of tissue injury by oxygen radicals, implications for neonatal disease. Acta Paediatr..

[24] Saugstad O.D. (2005). Oxidative stress in the newborn—A 30-year perspective. Neonatology.

[25] Toescu V., Nuttall S.L., Martin U., Kendall M.J., Dunne F. (2010). Oxidative stress and normal pregnancy. Clin. Endocrinol..

[26] Herrera E., Ortega-Senovilla H. (2010). Maternal lipid metabolism during normal pregnancy and its implications to fetal development. Clin. Lipidol..

[27] Mou D., Wang J., Liu H., Chen Y., Che L., Fang Z., Xu S., Lin Y., Feng B., Li J. (2018). Maternal methyl donor supplementation during gestation counteracts bisphenol A—Induced oxidative stress in sows and offspring. Nutrition.

[28] Langille M.G., Zaneveld J., Caporaso J.G., McDonald D., Knights D., Reyes J.A., Clemente J.C., Burkepile D.E., Thurber R.L.V., Knight R. (2013). Predictive functional profiling of microbial communities using 16S rRNA marker gene sequences. Nat. Biotechnol..

[29] Minelli A., Bellezza I., Conte C., Culig Z. (2009). Oxidative stress-related aging, A role for prostate cancer?. Biochim. Biophys. Acta.

[30] David M., Munaswamy V., Halappa R., Marigoudar S.R. (2008). Impact of sodium cyanide on catalase activity in the freshwater exotic carp, *Cyprinus carpio* (Linnaeus). Pestic. Biochem. Physiol..

[31] Finkel T., Holbrook N.J. (2000). Oxidants, oxidative stress and the biology of ageing. Nature.

[32] Ren W., Yin Y., Liu G., Yu X., Li Y., Yang G., Li T., Wu G. (2012). Effect of dietary arginine supplementation on reproductive performance of mice with porcine circovirus type 2 infection. Amino Acids.

[33] Li H., Song F., Duan L.-R., Sheng J.-J., Xie Y.-H., Yang Q., Chen Y., Dong Q.Q., Zhang B.L., Wang S.W. (2016). Paeonol and danshensu combination attenuates apoptosis in myocardial infarcted rats by inhibiting oxidative stress, Roles of Nrf2/HO-1 and PI3K/Akt pathway. Sci. Rep. UK.

[34] Yi D., Hou Y., Wang L., Ding B., Yang Z., Li J., Long M., Liu Y., Wu G. (2014). Dietary N-acetylcysteine supplementation alleviates liver injury in lipopolysaccharide-challenged piglets. Brit. J. Nutr..

[35] Cheng L., Jin Z., Zhao R., Ren K., Deng C., Yu S. (2015). Resveratrol attenuates inflammation and oxidative stress induced by myocardial ischemia-reperfusion injury, role of Nrf2/ARE pathway. Int. J. Clin. Exp. Med..

[36] Chen J., Yu B., Chen D., Huang Z., Mao X., Zheng P., Yu J., Luo J., He J. (2018). Chlorogenic acid improves intestinal barrier functions by suppressing mucosa inflammation and improving antioxidant capacity in weaned pigs. J. Nutr. Biochem..

[37] Kim Y.M., Pae H.O., Park J.E., Lee Y.C., Woo J.M., Kim N.H., Choi Y.K., Lee B.-S., Kim S.R., Chung H.T. (2011). Heme oxygenase in the regulation of vascular biology, from molecular mechanisms to therapeutic opportunities. Antioxid. Redox Signal..

[38] Al-Sadi R., Boivin M., Ma T. (2009). Mechanism of cytokine modulation of epithelial tight junction barrier. Front. Biosci..

[39] Capaldo C.T., Nusrat A. (2009). Cytokine regulation of tight junctions. Biochim. Biophys. Acta Biomembr..

[40] Maes M., Scharpé S., Meltzer H.Y., Bosmans E., Suy E., Calabrese J., Cosyns P. (1993). Relationships between interleukin-6 activity, acute phase proteins, and function of the hypothalamic-pituitary-adrenal axis in severe depression. Psychiatry Res..

[41] Kimura A., Kishimoto T. (2010). IL-6, regulator of Treg/Th17 balance. Eur. J. Immunol..

[42] Gitto E., Romeo C., Reiter R.J., Impellizzeri P., Pesce S., Basile M., Antonuccio P., Trimarchi G., Gentile C., Barberi I. (2004). Melatonin reduces oxidative stress in surgical neonates. J. Pediatr. Surg..

[43] Van Antwerp D.J., Martin S.J., Kafri T., Green D.R., Verma I.M. (1996). Suppression of TNF-α-induced apoptosis by NF-κB. Science.

[44] Cheng C., Wei H., Xu C., Xie X., Jiang S., Peng J. (2018). Maternal soluble fiber diet during pregnancy changes the intestinal microbiota, improves growth performance, and reduces intestinal permeability in piglets. Appl. Environ. Microbiol..

[45] Krishnamurthy V.M.R., Wei G., Baird B.C., Murtaugh M., Chonchol M.B., Raphael K.L., Greene T., Beddhu S. (2012). High dietary fiber intake is associated with decreased inflammation and all-cause mortality in patients with chronic kidney disease. Kidney Int..

[46] King D.E. (2005). Dietary fiber, inflammation, and cardiovascular disease. Mol. Nutr. Food Res..

[47] Segovia S.A., Vickers M.H., Gray C., Reynolds C.M. (2014). Maternal obesity, inflammation, and developmental programming. BioMed Res. Int..

[48] Reuter S., Gupta S.C., Chaturvedi M.M., Aggarwal B.B. (2010). Oxidative stress, inflammation, and cancer, how are they linked?. Free Radic. Biol. Med..

[49] Ma T.Y., Iwamoto G.K., Hoa N.T., Akotia V., Pedram A., Boivin M.A., Said H.M. (2004). TNF-α-induced increase in intestinal epithelial tight junction permeability requires NF-κB activation. Am. J. Physiol. Gastrointest. Liver Physiol..

[50] Li Y., Zhang L., Liu H., Yang Y., He J., Cao M., Yang M., Zhong W., Lin Y., Zhuo Y. (2019). Effects of the ratio of insoluble fiber to soluble fiber in gestation diets on sow performance and offspring intestinal development. Animals.

[51] Hakansson A., Molin G. (2011). Gut microbiota and inflammation. Nutrients.

[52] Holscher H.D. (2017). Dietary fiber and prebiotics and the gastrointestinal microbiota. Gut Microbes.

[53] Turnbaugh P.J., Ley R.E., Mahowald M.A., Magrini V., Mardis E.R., Gordon J.I. (2006). An obesity-associated gut microbiome with increased capacity for energy harvest. Nature.

[54] Tsatsaronis J.A., Walker M.J., Sanderson-Smith M.L. (2014). Host responses to group a streptococcus, cell death and inflammation. PLoS Pathog..

[55] Heuvelin E., Lebreton C., Grangette C., Pot B., Cerf-Bensussan N., Heyman M. (2009). Mechanisms involved in alleviation of intestinal inflammation by *Bifidobacterium breve* soluble factors. PLoS ONE.

[56] Miyazaki Y., Kamiya S., Hanawa T., Fukuda M., Kawakami H., Takahashi H., Yokota H. (2010). Effect of probiotic bacterial strains of *Lactobacillus*, *Bifidobacterium*, and *Enterococcus* on enteroaggregative *Escherichia coli*. J. Infect. Chemother..

[57] Park J.S., Lee E.J., Lee J.C., Kim W.K., Kim H.S. (2007). Anti-inflammatory effects of short chain fatty acids in IFN-γ-stimulated RAW 264.7 murine macrophage cells, Involvement of NF-κB and ERK signaling pathways. Int. Immunopharmacol..

[58] Tilg H., Moschen A.R. (2010). Evolution of inflammation in nonalcoholic fatty liver disease, the multiple parallel hits hypothesis. Hepatology.

[59] Huang W., Guo H.L., Deng X., Zhu T.T., Xiong J.F., Xu Y.H., Xu Y. (2017). Short-chain fatty acids inhibit oxidative stress and inflammation in mesangial cells induced by high glucose and lipopolysaccharide. Exp. Clin. Endocrinol. Diabetes.

[60] Yaku K., Enami Y., Kurajyo C., Matsui-Yuasa I., Konishi Y., Kojima-Yuasa A. (2012). The enhancement of phase 2 enzyme activities by sodium butyrate in normal intestinal epithelial cells is associated with Nrf2 and p53. Mol. Cell. Biochem..

[61] Place R.F., Noonan E.J., Giardina C. (2005). HDAC inhibition prevents NF-κB activation by suppressing proteasome activity, down-regulation of proteasome subunit expression stabilizes IκBα. Biochem. Pharmacol..

[62] Aagaard K., Ma J., Antony K.M., Ganu R., Petrosino J., Versalovic J. (2014). The placenta harbors a unique microbiome. Sci. Transl. Med..

[63] Leblois J., Massart S., Li B., Wavreille J., Bindelle J., Everaert N. (2017). Modulation of piglets’ microbiota, differential effects by a high wheat bran maternal diet during gestation and lactation. Sci. Rep. UK.

[64] Collado M.C., Rautava S., Aakko J., Isolauri E., Salminen S. (2016). Human gut colonisation may be initiated in utero by distinct microbial communities in the placenta and amniotic fluid. Sci. Rep. UK.

[65] Dominguez-Bello M.G., Costello E.K., Contreras M., Magris M., Hidalgo G., Fierer N., Knight R. (2010). Delivery mode shapes the acquisition and structure of the initial microbiota across multiple body habitats in newborns. Proc. Natl. Acad. Sci. USA.

[66] Gohir W., Whelan F.J., Surette M.G., Moore C., Schertzer J.D., Sloboda D.M. (2015). Pregnancy-related changes in the maternal gut microbiota are dependent upon the mother’s periconceptional diet. Gut Microbes.

[67] Ma J., Prince A.L., Bader D., Hu M., Ganu R., Baquero K., Blundell P., Harris R.A., Frias A.E., Grove K.L. (2014). High-fat maternal diet during pregnancy persistently alters the offspring microbiome in a primate model. Nat. Commun..

[68] Rolhion N., Darfeuille-Michaud A. (2007). Adherent-invasive *Escherichia coli* in inflammatory bowel disease. Inflamm. Bowel Dis..

[69] Wang Z., Xiao G., Yao Y., Guo S., Lu K., Sheng Z. (2006). The role of bifidobacteria in gut barrier function after thermal injury in rats. J. Trauma.

[70] Munoz-Price L.S., Weinstein R.A. (2008). Acinetobacter infection. New Engl. J. Med..

[71] Joly-Guillou M.L. (2005). Clinical impact and pathogenicity of *Acinetobacter*. Clin. Microbiol. Infect..

[72] Peleg A.Y., Seifert H., Paterson D.L. (2008). *Acinetobacter baumannii*, emergence of a successful pathogen. Clin. Microbiol. Rev..

[73] Zhou P., Zhao Y., Zhang P., Li Y., Gui T., Wang J., Jin C., Che L., Li J., Lin Y. (2017). Microbial mechanistic insight into the role of inulin in improving maternal health in a pregnant sow model. Front. Microbiol..

