# Supplementary material for: Maternal Dietary Fiber Composition during Gestation Induces Changes in Offspring Antioxidative Capacity, Inflammatory Response, and Gut Microbiota in a Sow Model
Source: Int J Mol Sci. 2019 Dec 19;21(1):31. doi: 10.3390/ijms21010031 (PMC6981455; doi:10.3390/ijms21010031)
Supplement: Supplementary file 1 [file ijms-21-00031-s001.pdf]

Table S1. Analysis of ANOSIM to access beta diversity of sow fecal microbiota on day 110 of pregnancy

Group-Pair	R-Value	*p* value
R1 *vs* R2	-0.009	0.484
R1 *vs* R3	0.326	0.006
R1 *vs* R4	0.270	0.003
R2 *vs* R3	0.213	0.010
R2 *vs* R4	0.104	0.090
R3 *vs* R4	0.024	0.331

R1, R2, R3 and R4 were diets in which the ratios of insoluble to soluble fiber were 3.89, 5.59, 9.12 and 12.81, respectively. $p < 0.05$ was considered statistically significant. n=6 for each treatment.

Table S2. Effects of the ratio of insoluble to soluble fiber in gestation diets of sows on the relative abundance of fecal microbiota in genus level

Taxonomy, %	Treatments				*p* value
	R1	R2	R3	R4	
Streptococcus	7.88±0.98^{b}	7.51±0.84^{b}	12.10±1.09^{a}	10.34±0.56^{a}	0.049
Clostridium_sensu_stricto_1	8.50±0.93	8.61±1.19	8.53±0.79	9.03±1.54	0.880
Prevotella_9	4.65±1.44	2.19±0.56	5.04±2.30	3.42±1.04	0.224
Prevotellaceae_NK3B31_group	3.28±0.34	3.06±0.37	2.90±0.65	5.39±1.81	0.092
Lactobacillus	4.40±1.08	3.06±0.59	3.69±0.50	4.69±1.40	0.429
Treponema_2	4.30±0.86^{ab}	4.84±0.63^{a}	4.65±0.90^{a}	1.93±0.57^{b}	0.015
Succinivibrio	1.09±0.29^{ab}	1.57±0.36^{ab}	0.89±0.19^{b}	2.87±0.74^{a}	0.011
Methanobrevibacter	2.18±0.31	1.39±0.33	0.95±0.37	1.66±0.33	0.127
Prevotellaceae_UCG-001	1.78±0.60	1.72±0.38	1.33±0.57	0.80±0.32	0.295
Lachnospiraceae_XPB1014_group	1.34±0.10	1.61±0.23	2.09±0.30	1.65±0.38	0.296
Ruminococcaceae_UCG-005	1.98±0.13^{b}	2.63±0.08^{a}	2.65±0.19^{a}	2.73±0.24^{a}	0.007
Megasphaera	0.55±0.12	0.42±0.07	0.26±0.05	0.70±0.28	0.052
Turicibacter	1.70±0.17	2.00±0.31	1.63±0.21	1.61±0.26	0.635
Rikenellaceae_RC9_gut_group	2.95±0.28	2.96±0.37	3.02±0.23	2.77±0.46	0.967
Ruminococcaceae_UCG-002	1.59±0.14	1.63±0.08	1.52±0.19	1.27±0.17	0.337
Prevotella_1	2.04±0.56^{a}	0.94±0.55^{ab}	0.49±0.06^{b}	0.45±0.10^{b}	0.003
Bifidobacterium	0.76±0.25^{ab}	1.36±0.58^{a}	0.41±0.09^{b}	0.31±0.06^{b}	0.006
Terrisporobacter	1.49±0.11	1.79±0.32	1.45±0.11	1.43±0.26	0.585
Ruminococcaceae_NK4A214_group	1.75±0.13	1.79±0.10	1.64±0.19	1.32±0.16	0.105
Alloprevotella	1.13±0.16	1.04±0.13	1.13±0.20	1.73±0.46	0.144
Anaerovibrio	0.54±0.13	0.88±0.50	0.55±0.09	0.84±0.25	0.434
Erysipelotrichaceae_UCG-002	0.38±0.19^{a}	0.13±0.03^{ab}	0.08±0.02^{b}	0.07±0.02^{b}	0.004
Oscillospira	0.92±0.14	1.45±0.35	1.06±0.09	1.32±0.24	0.130
Phascolarctobacterium	0.63±0.18	0.46±0.05	0.50±0.06	0.75±0.18	0.262
Ruminococcus_1	1.09±0.19^{a}	1.38±0.32^{a}	0.87±0.10^{ab}	0.52±0.08^{b}	0.002
Prevotellaceae_UCG-003	1.18±0.08	1.14±0.12	1.68±0.26	1.28±0.12	0.058
Ruminococcaceae_UCG-014	0.85±0.08^{a}	0.91±0.05^{a}	0.71±0.15^{a}	0.41±0.08^{b}	0.002
Bacteroides	0.37±0.06^{b}	0.41±0.07^{b}	0.72±0.10^{ab}	0.88±0.23^{a}	0.007
[Eubacterium]_coprostanoligenes_group	1.02±0.09^{a}	0.91±0.03^{ab}	0.93±0.15^{ab}	0.68±0.06^{b}	0.046
Parabacteroides	0.94±0.14^{ab}	1.19±0.14^{ab}	0.80±0.07^{b}	1.35±0.12^{a}	0.013
Romboutsia	0.57±0.05	0.73±0.14	0.55±0.07	0.52±0.10	0.391

Christensenellaceae_R-7_group	0.74±0.07	0.87±0.05	0.81±0.06	0.74±0.08	0.364
Prevotella_7	0.21±0.05	0.20±0.05	0.25±0.18	0.27±0.13	0.932
dgA-11_gut_group	0.62±0.14	0.48±0.04	0.49±0.05	0.35±0.07	0.095
Campylobacter	0.21±0.01^{b}	0.36±0.07^{ab}	0.40±0.08^{ab}	0.50±0.14^{a}	0.030

[1]R1, R2, R3 and R4 were diets in which the ratios of insoluble to soluble fiber were 3.89, 5.59, 9.12 and 12.81, respectively.

Values are mean ± standard error (n=6). $^{a\text{-}b}$Means with different superscripts within a row differ ($P < 0.05$).

Table S3. Analysis of ANOSIM to access beta diversity of neonatal piglet colonic microbiota

Group-Pair	R-Value	*p* value
R1 *vs* R2	-0.033	0.275
R1 *vs* R3	0.607	0.013
R1 *vs* R4	0.554	0.005
R2 *vs* R3	0.457	0.002
R2 *vs* R4	0.354	0.024
R3 *vs* R4	-0.051	0.578

R1, R2, R3 and R4 were diets in which the ratios of insoluble to soluble fiber were 3.89, 5.59, 9.12 and 12.81, respectively. $P < 0.05$ was considered statistically significant. n=6 for each treatment.

Table S4. Composition of the experimental diets during pregnancy (as-fed basis)

Items	Treatments			
	R1	R2	R3	R4
Ingredient，%				
Corn	71.50	71.50	71.50	71.50
Dehulled soybean meal	10.20	10.20	10.20	10.20
Wheat bran	13.00	13.00	13.00	13.00
Cellulose	-	1.00	2.00	2.50
Inulin	2.50	1.50	0.50	-
L-lysine HCl	0.17	0.17	0.17	0.17
DL-methionine	0.01	0.01	0.01	0.01
L-threonine	0.04	0.04	0.04	0.04
Calcium carbonate	0.97	0.97	0.97	0.97
Dicalcium phosphate	0.52	0.52	0.52	0.52
Salt	0.40	0.40	0.40	0.40
Choline chloride	0.14	0.14	0.14	0.14
Vitamin premix[1]	0.05	0.05	0.05	0.05
Mineral premix[2]	0.50	0.50	0.50	0.50
Total	100.00	100.00	100.00	100.00
Calculated analysis				
Digestible energy, Mcal/kg	3.05	3.05	3.05	3.05
Crude protein, %	12.13	12.13	12.13	12.13
Crude fat, %	3.22	3.22	3.22	3.22
Crude fiber, %	4.83	4.83	4.83	4.83
Soluble fiber (SF), %	3.87	2.87	1.87	1.37
Insoluble fiber (ISF), %	15.04	16.04	17.04	17.54
Ratio of ISF to SF	3.89	5.59	9.12	12.81
Dietary fiber, %	18.91	18.91	18.91	18.91
Ca, %	0.55	0.55	0.55	0.55
Available P, %	0.24	0.24	0.24	0.24
Lysine, %	0.55	0.55	0.55	0.55
Methionine, %	0.18	0.18	0.18	0.18
Threonine, %	0.41	0.41	0.41	0.41
Tryptophan, %	0.12	0.12	0.12	0.12

[1]Provided per kg of diet: vitamin A 7,500 IU, vitamin D_3 5,000 IU, vitamin E 37.5 IU, vitamin K_3 5 mg, vitamin B_1 5 mg, vitamin B_2 12.5 mg, vitamin B_6 7.5 mg, vitamin B_{12} 0.05 mg, biotin 0.2 mg, niacin 50 mg, folic acid 2.5 mg, D-calcium pantothenate 25 mg.

[2]Provided per kg of diet: Cu 10 mg ($CuSO_4$), Fe 100 mg ($FeSO_4$), I 0.6 mg (KI), Zn 100 mg ($ZnSO_4$), Mn 30 mg ($MnSO_4$), Se 0.25 mg (Na_2SeO_3).

Table S5. Primer sequences used for quantitative real-time PCR

Genes	Gene bank No.	Primer sequences [1] (5'-3')	Size, bp
β-actin	AY550069.1	F: AACTGGAACGGTGAAGGTGA<br>R: CTTTTGGAAAGGCAGGGACT	174
Nrf2	XM_021075133.1	F: GCCCCTGGAAGCGTTAAAC<br>R: GGACTGTATCCCCAGAAGGTTGT	67
HO-1	NM_001004027.1	F: AGCTGTTTCTGAGCCTCCAA<br>R: CAAGACGGAAACACGAGACA	130
NF-κB	NM_001114281.1	F: AGTACCCTGAGGCTATAACTCGC<br>R: TCCGCAATGGAGGAGAAGTC	133

[1] F, forward; R, reverse.
